# Supplementary material for: Multi‐dataset identification of innovative feature genes and molecular mechanisms in keratoconus
Source: J Cell Mol Med. 2024 Sep 19;28(18):e70079. doi: 10.1111/jcmm.70079 (PMC11412914; doi:10.1111/jcmm.70079)
Supplement: Supplementary file 2 — Material S2. [file JCMM-28-e70079-s002.zip › 7.GSEA analysis/3.DBNDD1/KEGG_PARKINSONS_DISEASE.html]

Details for gene set KEGG\_PARKINSONS\_DISEASE[GSEA]

|  || Dataset | DBNDD1.group.cls#h\_versus\_l |
| Phenotype | group.cls#h\_versus\_l |
| Upregulated in class | h |
| GeneSet | KEGG\_PARKINSONS\_DISEASE |
| Enrichment Score (ES) | 0.20653434 |
| Normalized Enrichment Score (NES) | 0.88428164 |
| Nominal p-value | 0.5618449 |
| FDR q-value | 1.0 |
| FWER p-Value | 1.0 |
Table: GSEA Results Summary

  

Fig 1: Enrichment plot: KEGG\_PARKINSONS\_DISEASE      
 Profile of the Running ES Score & Positions of GeneSet Members on the Rank Ordered List

  

| SYMBOL | TITLE | RANK IN GENE LIST | RANK METRIC SCORE | RUNNING ES | CORE ENRICHMENT || 1 | SLC18A2 | na | 1150 | 0.367 | 0.0473 | Yes |
| 2 | LRRK2 | na | 2100 | 0.293 | 0.0842 | Yes |
| 3 | SLC25A31 | na | 2127 | 0.291 | 0.1473 | Yes |
| 4 | UCHL1 | na | 2503 | 0.272 | 0.1962 | Yes |
| 5 | APAF1 | na | 8227 | 0.099 | 0.0528 | Yes |
| 6 | SLC18A1 | na | 8430 | 0.096 | 0.0680 | Yes |
| 7 | COX7A2L | na | 8857 | 0.088 | 0.0750 | Yes |
| 8 | COX6A2 | na | 8938 | 0.087 | 0.0917 | Yes |
| 9 | COX4I2 | na | 9035 | 0.085 | 0.1076 | Yes |
| 10 | SLC6A3 | na | 9787 | 0.074 | 0.1022 | Yes |
| 11 | COX6B2 | na | 10294 | 0.067 | 0.1024 | Yes |
| 12 | NDUFB5 | na | 10463 | 0.065 | 0.1117 | Yes |
| 13 | GPR37 | na | 11039 | 0.058 | 0.1078 | Yes |
| 14 | TH | na | 11060 | 0.057 | 0.1198 | Yes |
| 15 | NDUFC2 | na | 11325 | 0.054 | 0.1241 | Yes |
| 16 | UBE2J1 | na | 11382 | 0.054 | 0.1343 | Yes |
| 17 | UBE2J2 | na | 11546 | 0.052 | 0.1409 | Yes |
| 18 | NDUFV3 | na | 11702 | 0.050 | 0.1474 | Yes |
| 19 | NDUFAB1 | na | 12930 | 0.039 | 0.1205 | Yes |
| 20 | NDUFB1 | na | 13049 | 0.038 | 0.1254 | Yes |
| 21 | NDUFS6 | na | 13068 | 0.038 | 0.1331 | Yes |
| 22 | PINK1 | na | 13220 | 0.036 | 0.1368 | Yes |
| 23 | CASP3 | na | 13339 | 0.035 | 0.1411 | Yes |
| 24 | NDUFB10 | na | 13393 | 0.035 | 0.1473 | Yes |
| 25 | UQCR11 | na | 13449 | 0.035 | 0.1533 | Yes |
| 26 | NDUFB8 | na | 13532 | 0.034 | 0.1584 | Yes |
| 27 | NDUFB7 | na | 13785 | 0.032 | 0.1580 | Yes |
| 28 | COX8A | na | 13890 | 0.031 | 0.1618 | Yes |
| 29 | SDHC | na | 14109 | 0.029 | 0.1619 | Yes |
| 30 | NDUFA9 | na | 14400 | 0.027 | 0.1595 | Yes |
| 31 | NDUFA4L2 | na | 14523 | 0.026 | 0.1617 | Yes |
| 32 | NDUFS1 | na | 14531 | 0.026 | 0.1673 | Yes |
| 33 | SLC25A6 | na | 14804 | 0.024 | 0.1648 | Yes |
| 34 | NDUFS4 | na | 14861 | 0.024 | 0.1684 | Yes |
| 35 | NDUFA10 | na | 14952 | 0.023 | 0.1709 | Yes |
| 36 | NDUFS2 | na | 15047 | 0.022 | 0.1731 | Yes |
| 37 | PPID | na | 15089 | 0.022 | 0.1768 | Yes |
| 38 | SLC25A5 | na | 15264 | 0.021 | 0.1763 | Yes |
| 39 | COX6B1 | na | 15292 | 0.021 | 0.1801 | Yes |
| 40 | UBE2L3 | na | 15321 | 0.020 | 0.1838 | Yes |
| 41 | COX5B | na | 15394 | 0.020 | 0.1861 | Yes |
| 42 | COX7B2 | na | 15452 | 0.020 | 0.1887 | Yes |
| 43 | COX7B | na | 15536 | 0.019 | 0.1905 | Yes |
| 44 | NDUFS8 | na | 15627 | 0.018 | 0.1919 | Yes |
| 45 | COX7A2 | na | 15716 | 0.018 | 0.1933 | Yes |
| 46 | NDUFA1 | na | 15785 | 0.017 | 0.1951 | Yes |
| 47 | NDUFC1 | na | 15837 | 0.017 | 0.1973 | Yes |
| 48 | NDUFA7 | na | 15872 | 0.017 | 0.2000 | Yes |
| 49 | UBE2L6 | na | 15975 | 0.016 | 0.2006 | Yes |
| 50 | UQCR10 | na | 16010 | 0.016 | 0.2031 | Yes |
| 51 | NDUFB3 | na | 16011 | 0.016 | 0.2065 | Yes |
| 52 | SDHD | na | 16304 | 0.014 | 0.2011 | No |
| 53 | NDUFA8 | na | 16485 | 0.013 | 0.1987 | No |
| 54 | NDUFB2 | na | 16493 | 0.013 | 0.2013 | No |
| 55 | UQCRC1 | na | 16639 | 0.012 | 0.1997 | No |
| 56 | NDUFS3 | na | 16717 | 0.011 | 0.1999 | No |
| 57 | UQCRC2 | na | 16785 | 0.011 | 0.2003 | No |
| 58 | COX4I1 | na | 16804 | 0.011 | 0.2022 | No |
| 59 | COX7C | na | 17085 | 0.009 | 0.1960 | No |
| 60 | NDUFS7 | na | 17112 | 0.009 | 0.1972 | No |
| 61 | NDUFA4 | na | 17126 | 0.009 | 0.1987 | No |
| 62 | NDUFB9 | na | 17132 | 0.009 | 0.2004 | No |
| 63 | UQCRQ | na | 17160 | 0.008 | 0.2014 | No |
| 64 | COX6C | na | 17183 | 0.008 | 0.2026 | No |
| 65 | NDUFA3 | na | 17211 | 0.008 | 0.2036 | No |
| 66 | VDAC3 | na | 17362 | 0.007 | 0.2008 | No |
| 67 | NDUFA5 | na | 17695 | 0.005 | 0.1923 | No |
| 68 | COX5A | na | 17706 | 0.005 | 0.1931 | No |
| 69 | CYC1 | na | 17708 | 0.005 | 0.1942 | No |
| 70 | NDUFV1 | na | 17902 | 0.003 | 0.1893 | No |
| 71 | NDUFV2 | na | 18101 | 0.002 | 0.1841 | No |
| 72 | UQCRH | na | 18129 | 0.002 | 0.1837 | No |
| 73 | SDHB | na | 18300 | 0.001 | 0.1789 | No |
| 74 | NDUFS5 | na | 18515 | -0.001 | 0.1729 | No |
| 75 | SLC25A4 | na | 18531 | -0.001 | 0.1727 | No |
| 76 | UBE2G2 | na | 18860 | -0.003 | 0.1640 | No |
| 77 | NDUFA2 | na | 18897 | -0.004 | 0.1638 | No |
| 78 | COX6A1 | na | 18979 | -0.004 | 0.1624 | No |
| 79 | NDUFB4 | na | 19246 | -0.006 | 0.1561 | No |
| 80 | UQCRFS1 | na | 19283 | -0.007 | 0.1566 | No |
| 81 | UQCRB | na | 19342 | -0.007 | 0.1565 | No |
| 82 | CASP9 | na | 19881 | -0.012 | 0.1436 | No |
| 83 | CYCS | na | 20083 | -0.014 | 0.1408 | No |
| 84 | NDUFA6 | na | 20241 | -0.015 | 0.1396 | No |
| 85 | PARK7 | na | 20597 | -0.018 | 0.1333 | No |
| 86 | UBA7 | na | 20895 | -0.021 | 0.1293 | No |
| 87 | VDAC2 | na | 21354 | -0.025 | 0.1217 | No |
| 88 | COX7A1 | na | 21665 | -0.029 | 0.1190 | No |
| 89 | UBB | na | 21781 | -0.030 | 0.1222 | No |
| 90 | NDUFB6 | na | 21907 | -0.031 | 0.1254 | No |
| 91 | SDHA | na | 22031 | -0.032 | 0.1290 | No |
| 92 | UBA1 | na | 22336 | -0.035 | 0.1280 | No |
| 93 | VDAC1 | na | 22895 | -0.042 | 0.1211 | No |
| 94 | UBE2G1 | na | 23179 | -0.045 | 0.1229 | No |
| 95 | HTRA2 | na | 23274 | -0.046 | 0.1304 | No |
| 96 | COX8C | na | 27951 | -0.130 | 0.0239 | No |
| 97 | SNCA | na | 32335 | -0.284 | -0.0404 | No |
| 98 | SNCAIP | na | 34338 | -0.499 | 0.0113 | No |
Table: GSEA details [plain text format]

  

Fig 2: KEGG\_PARKINSONS\_DISEASE      
 Blue-Pink O' Gram in the Space of the Analyzed GeneSet

  

Fig 3: KEGG\_PARKINSONS\_DISEASE: Random ES distribution      
 Gene set null distribution of ES for **KEGG\_PARKINSONS\_DISEASE**
